# Comparative Genomics of an Emerging Multidrug-Resistant *bla*_NDM_-Carrying ST182 Lineage in *Enterobacter cloacae* Complex

**DOI:** 10.3390/antibiotics13060535

**Published:** 2024-06-08

**Authors:** Angeliki Mavroidi, Elisavet Froukala, Athanasios Tsakris

**Affiliations:** 1 Department of Microbiology, Faculty of Medicine, General University Hospital of Patras, 26504 Patras, Greece; amavroidi@live.com; 2 Department of Microbiology, Medical School, University of Athens, 11527 Athens, Greece; elisavetfrou@gmail.com

**Keywords:** *Enterobacter cloacae* complex, *E. hormaechei*, NDM carbapenemase, MLST, WGS

## Abstract

Background: *Enterobacter cloacae*, *E. hormaechei* and related subspecies remain the most clinically relevant among the *Enterobacter cloacae* complex (ECC). Carbapenemase-producing ECC strains are increasingly identified in hospital-acquired infections and usually belong to four main multilocus sequence types (MLST STs) named ST114, ST93, ST90 and ST78. Instead, ST182 has been sporadically reported among *E. hormaechei* strains, and recently, outbreaks of *bla*_NDM_-producing ST182 clonal strains have emerged. Herein, we aimed to investigate the presence of ST182 and explore its evolution and modes of *bla*_NDM_ acquisition. Methods: A phylogenetic analysis of 646 MLST STs identified among 4685 *E. hormaechei* whole-genome sequencing (WGS) assemblies deposited in public repositories was performed, as well as an in silico comparative and phylogenomic analyses for 55 WGS assemblies of ST182. *bla*_NDM_-harboring contigs were also compared to published plasmid sequences. Results: ST182 *E. hormaechei* strains were recovered from patients on five continents during 2011–2021. They were divided into three major genomic clusters, comprising a separate clonal complex with six other STs. In 30 out of 55 ST182 WGS assemblies, *bla*_NDM_-harboring structures were identified that were similar to the plasmids predominant in Gram-negative bacteria, harboring resistance genes to multiple antibiotic classes and virulence genes. No associations between the genomic clusters and the country/continent of isolation or the presence and the plasmid types of the *bla*_NDM_-harboring contigs were observed. Conclusions: Our findings show that ST182 *E. hormaechei* strains have been identified in the past decade worldwide; 54.5% of them carried diverse *bla*_NDM_ genetic structures, suggesting recent acquisition of the *bla*_NDM_ alleles. Thus, *bla*_NDM_-harboring ST182 is an emerging multidrug-resistant and virulent lineage in ECC strains that requires close monitoring.

## 1. Introduction

*Enterobacter cloacae* complex (ECC) species are often recognized as the causative agents of hospital-acquired infections, such as pneumonia, urinary tract and soft tissue infections, septicemia, and meningitis [1]. Among them, *E. cloacae* and *E. hormaechei* are the most frequently identified in clinical specimens from hospitalized patients [1,2]. *Enterobacter* species are considered members of the ESKAPE (*Enterococcus faecium*, *Staphylococcus aureus*, *Klebsiella pneumoniae*, *Acinetobacter baumannii*, *Pseudomonas aeruginosa* and *Enterobacter* species) group of pathogens, exhibiting resistance to most common antibacterial treatments [3]. Multidrug-resistant ECC isolates are often reported worldwide [1,3,4,5,6,7,8]. More specifically, ECC isolates are intrinsically resistant to first- and second-generation cephalosporins due to the presence of chromosomally encoded AmpC (class C) β-lactamases, whereas resistance to multiple antibiotic classes has been associated mostly with overexpression of efflux pumps and the acquisition via mobile gene elements (MGEs) of resistance genes from other species, e.g., extended-spectrum β-lactamase (ESBL), carbapenemase, aminoglycoside and quinolone resistance genes [3,4,5,6,7,8].

Carbapenemase-producing ECC strains are now reported in all WHO health regions [1,3,4,5,6,7,8] and may contain class A and class D carbapenemases, which are serine carbapenemases and class B metallo-β-lactamases (MBLs) [1,4]. In particular, MDR *bla*_NDM_-producing ECC strains have recently emerged, causing nosocomial outbreaks in several countries [8]. New Delhi metallo-beta-lactamase (NDM) is an MBL-type carbapenemase, which is able to hydrolyze a wide range of β-lactams, including carbapenems, but not monobactams [5,6,8]. Furthermore, NDM is not inactivated by most of the recently developed β-lactamase inhibitors [5,6]. NDM-1 was first reported in a *K. pneumoniae* strain recovered from a urinary culture on 9 January 2008 of a Swedish patient with a history of hospitalization in New Delhi, India [5]. Thereafter, it has spread rapidly and 24 NDM variants have been identified in various species of Gram-negative bacteria, such as Enterobacterales, *Acinetobacter* spp. and *Pseudomonas* spp., from clinical specimens worldwide [1,2,3,4,5,6,8]. Among carbapenemase-producing ECC strains, the most common lineage ECC multilocus sequence type (MLST) ST114 is globally distributed and associated not only with NDM-type carbapenemases but also with several other carbapenemases, such as VIM-1 MBL, class A KPC-2 and class D OXA-48 [1,3,4,5,6,7,8]. ST78 and ST171 have also been reported as two emerging lineages of carbapenem-resistant ECC, but strains of these lineages usually produce KPC rather than NDM carbapenemases [4]. Nonetheless, these STs comprise only a minority of STs found in ECC strains [4,5,6,7,8]. ECC strains have a very diverse clonal population structure, and there are neither well-defined international clones nor obvious associations with NDM-positive ECC strains [5,6,7,8].

Instead, ST182 ECC isolates have been sporadically reported worldwide from clinical specimens [4,5,6,7,8,9,10]. In Europe, an ST182 *bla*_NDM-4_-producing isolate from a Czech patient previously hospitalized in Sri Lanka was reported for the first time in 2012 [11]. Later on, in 2016, ST182 caused an outbreak of *bla*_NDM-4_-producing ECC strains in the same country [12]. Additionally, a recent study of multidrug-resistant ECC isolates from Lebanon has shown that ST182 was the second most frequent ST accounting for 10.4% of the ECC isolates [13]. Lately, the largest European dissemination of ECC NDM producers has been reported in Greece and the outbreak was caused by an ST182 clonal strain [14]. Whole-genome taxonomic analysis of two *bla*_NDM-1_-producing strains recovered during the outbreak (EC-ML559 of MLST ST182 and EC-ML621 of ST2143, a single-locus variant of ST182) revealed that both strains were assigned as *E. hormaechei* [15]. In silico prediction of components of the bacterial cell surface and genomic islands showed the presence of various virulence factors and resistance genes to several antimicrobial classes, as well as differences in the plasmids carrying β-lactamase genes [15].

In the present study, we aimed to investigate the presence of whole-genome sequencing (WGS) assemblies of ST182 *E. hormaechei* in public databases and explore their characteristics, geographic distribution and evolution. For this purpose, we have compared in silico the WGS assemblies of ECC isolates of ST182, including plasmid types, and antimicrobial resistance and virulence genes. Furthermore, the *bla*_NDM_-harboring contigs of the WGS assemblies of the isolates were compared with published plasmid sequences to explore the plausible modes of acquiring *bla*_NDM_ alleles.

## 2. Results

### 2.1. Bacterial Strains, Whole-Genome Sequences and Phylogenetic Analysis of ECC Isolates

**Figure 1:**
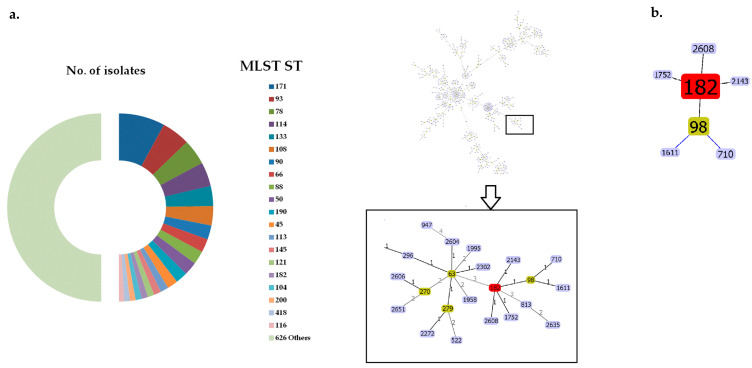
(**a**) Phylogenetic relationships of 646 MLST STs (*n* = 4685 isolates) retrieved by the Phyloviz version 2.0 software. The numbers of the allelic differences are shown on the lines of the branches of the phylogenetic tree. ST nodes colors denote: light green - group founder, light blue - common node, red – selected node (ST182). (**b**) Assignment of ST182 *E. hormaechei* into a clonal complex with ST98, ST710, ST1611, ST1752, ST2143 and ST2608 using the goeBURST algorithm.

The WGS assemblies retrieved from public databases have been obtained from *E. hormaechei* isolates (*n* = 4685), which belonged to 646 MLST STs. In this dataset, the most prevalent STs were ST171 (*n* = 396), ST93 (*n* = 244), ST78 (*n* = 220) and ST114 (*n* = 208), whereas ST182 (*n* = 55) ranked at position 16 (Figure 1a). By implementing the goeBURST algorithm and PHYLOViz analysis based on the MLST allelic profiles, the possible phylogenetic relationships between STs were obtained. Of the 646 MLST STs, 400 MLST STs (3953 isolates) were clustered into 74 CCs, whereas the remaining 246 STs (732 isolates) were singletons (i.e., each group comprised one ST) (Table S1). ST182 comprised a separate lineage in the phylogenetic tree, being in the same CC as ST98, ST710, ST1611, ST1752, ST2143 and ST2608 (Figure 1b).

**Figure 2:**
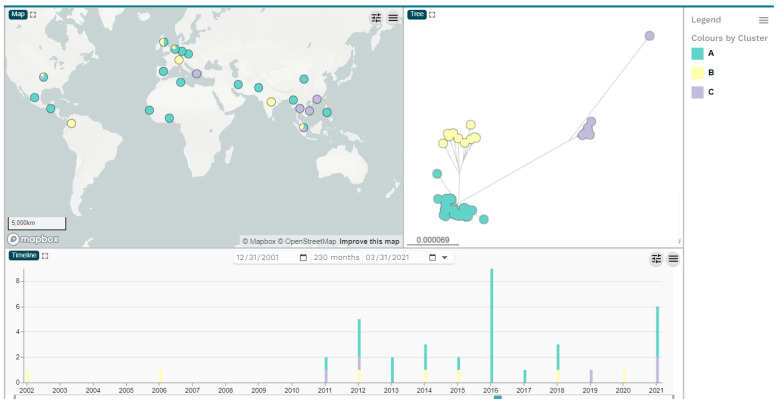
The geographical distribution, phylogenomic analysis and timeline of isolation of 55 ST182 *E. hormaechei* obtained with the Microreact web application (available at: https://microreact.org/).

Among the nucleotide sequences of the 55 ST182 *E. hormaechei* WGS assemblies, there were 4554 SNPs, and the overall mean distance was 0.1013 (Figure S1). Phylogenomic analysis of the WGS assemblies has revealed that the strains were distributed into three genomic clusters (sublineages); cluster A (*n* = 37), cluster B (*n* = 10) and cluster C (*n* = 8) [Figure 2, Figures S1 and S2; Table 1; Table S2]. The 55 ST182 WGS assemblies were collected mainly from Asia (*n* = 17), Europe (*n* = 17) and North America (*n* = 14) but also from Africa (*n* = 4) and South America (*n* = 3), while 30 of them carried *bla*_NDM_ genes (Table 1; Table S2). The first WGS assemblies of ST182 strains with no *bla*_NDM_ genes were identified in the United Kingdom collected in 2002 and 2006, which belonged to cluster B. No *bla*_NDM_ genes were identified in nine out of ten cluster B isolates, whereas one isolate collected from India carried *bla*_NDM-1_. Both *bla*_NDM_ carriers and strains with no *bla*_NDM_ genes were recovered annually from 2011 and onwards (Figure S3). 

### 2.2. In Silico Identification of Plasmids, Antimicrobial Resistance and Virulence Genes of the ECC NDM-Harboring Isolates

**Figure 3:**
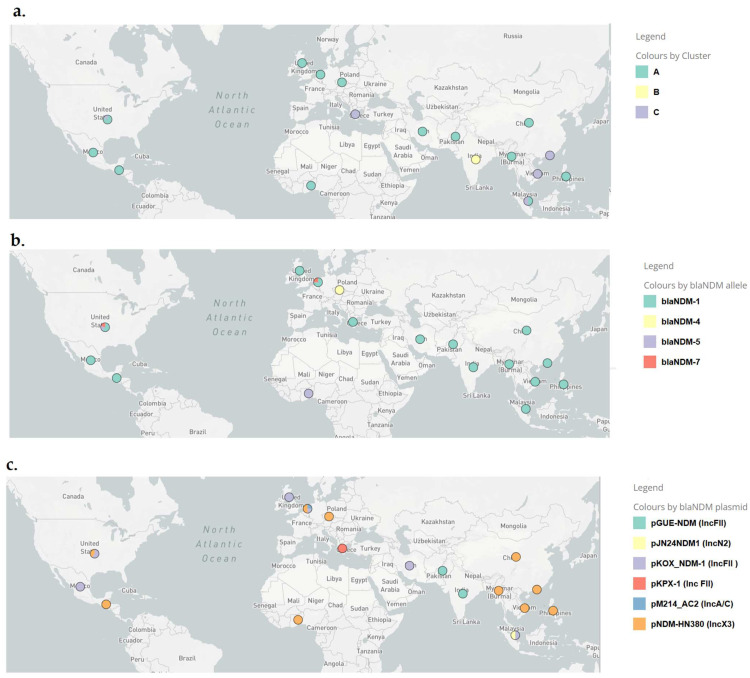
The geographical distribution of the (**a**) genomic clusters, (**b**) *bla*_NDM_ variants and (**c**) *bla*_NDM_ plasmid types of 30 ST182 *E.hormaechei bla*_NDM_ carriers obtained with the Microreact web application (available at: https://microreact.org/).

The characteristics and the predictions for the presence of plasmids, antimicrobial resistance and virulence genes of the 30 *bla*_NDM_-carrying WGS assemblies of ST182 are shown in Tables S3 and S4. All isolates were predicted to carry plasmids predominant in Gram-negative antibiotic-resistant strains, belonging to several incompatibility groups, such as IncX3, IncFII/IncFIB, IncHI2, IncHI2A, IncL, IncM, IncN, IncN3, IncR, IncX5, Col440I and Col440II replicon-type plasmids. No associations were observed between the country/continent of isolation or the presence of the *bla*_NDM_ plasmid types and the genetic clusters (Figure 3, Figure S2, Table S5).

All isolates were predicted to harbor antimicrobial resistance genes to multiple antibiotic classes and virulence genes. In more detail, four *bla*_NDM_ variants were identified: *bla*_NDM-1_ (*n* = 24), *bla*_NDM-4_ (*n* = 2), *bla*_NDM-5_ (*n* = 2) and *bla*_NDM-7_ (*n* = 2). Besides *bla*_NDM_, all carried the chromosomal AmpC-type (class C) cephalosporinase gene *bla*_ACT-16_ and several acquired β-lactamase genes, including *bla*_TEM-1_, *bla*_TEM-104_, *bla*_OXA-1_, *bla*_OXA-9_, *bla*_OXA-10_, *bla*_OXA-48,_ *bla*_CTX-M-3_, *bla*_CTX-M-9_, *bla*_CTX-M-14_, *bla*_CTX-M-15_, *bla*_DHA-1_, *bla*_KPC-2_, *bla*_SHV-12_, *bla*_LAP-2_, *bla*_SFO-1_ and *bla*_GES-5_. In addition to the β-lactamase genes, acquired genes conferring resistance to various antimicrobial classes were identified, including aminoglycosides (*aac(3)-IIa, aac(3)-Id, aac(3)-IId, aac(6’)-Ib, aac(6’)-Ib3, aac(6’)-IIc, aac(6’)-Ib-cr*, *aadA1*, *aadA2*, *aadA2b, aadA16*, *aph(3’)-Ia, aph(3’’)-Ib, aph(6)-Id, ant(2’’)-Ia, armA, rmtB, rmtC*), quinolones (*qnrA1, qnrB1, qnrB4, qnrB6, qnrB19, qnrS1, OqxA, OqxB*), chloramphenicol (*catA2, catB3*), trimethoprim (*dfrA12, dfrA14, dfrA19, dfrA27),* sulphonamides (*sul1, sul2*), macrolide–lincosamide–streptogramin B (MLS) (*mph(A), mph(E), ere(A), msr(E)*), tetracyclines (*tet(A), tet(D*), polymyxins *(mcr-9*), fosfomycin (*fosA*) and rifampicin (*ARR-3*). Moreover, resistance genes for quaternary ammonium compounds (*qacE*) and formaldehyde (*formA*) were also predicted.

Of the 30 *bla*_NDM_-harboring strains, 23 strains were predicted to harbor plasmidic sequences similar to the 139 kb *E. cloacae subsp. cloacae* ATCC13047 plasmid pECL_A (Table S4), which carries several virulence factor genes, such as two clusters of Type IV secretion system (T4SS) genes, associated with pathogenesis in plants and mammalian bacterial pathogens, and also multiple heavy metal resistance operons for copper, tellurium and mercury that are not conserved with other *Enterobacter* species but share notably high homology to *Cronobacter sakazakii*, *K. pneumoniae* and *E. coli* [16]. Additionally, the virulence genes *nlpI*, *terC, traT* and *mrkA, shiB, kpsM_K11* and *astA* were identified (Table S4). All WGS assemblies possessed the virulence gene *nlpI*, encoding the lipoprotein NlpI, which is involved in cell division, virulence and bacterial interaction with eukaryotic host cells [17]. All but one isolate (strain PEER1096 from India) possessed *terC,* which is one of the key proteins of the tellurite resistance gene operon (*ter*) involved in tellurite resistance phage inhibition, colicine resistance and pathogenicity [18]. Four strains also harbored the *traT* gene encoding the TraT protein, a cell-surface-exposed, outer membrane lipoprotein associated with resistance to the bactericidal activities of serum and prevention of self-mating of cells carrying identical or closely related conjugative plasmids [19]. The *mrkA* adhesion gene, which has been associated with biofilm formation in carbapenemase-producing *K. pneumoniae* [20], was present in three strains. Virulence genes found in pathogenic *E. coli* strains [21,22] were also predicted in three *bla*_NDM-1_- carriers; the *astA* gene in Biosamples SAMN25161196 and SAMN25161198 from the United States and the *kpsM_K11* gene in Biosample SAMN15904743 from China. Finally, the *shiB* gene, which has been found previously in the pathogenicity island SHI-2 (*Shigella* island 2) of *Shigella flexneri* [23], was predicted in a *bla*_NDM-1_ carrier (Biosample SAMEA8581547 from Pakistan).

### 2.3. Genetic Background of bla_NDM_ and Plasmid Analysis

In three ST182 strains (M515, MY196 and AZ 664), a *bla*_NDM-1_ gene was located on two different contigs of the WGS assemblies. BlastN comparisons of the *bla*_NDM_-harboring contigs revealed the presence of genetic structures showing 100% identities with regions of six different plasmid types; an IncX3 (pNDM-HN380), three different IncFII (pKOX_NDM-1, pGUE-NDM, pKPX-1), an IncA/C (pM214_AC2) and an IncN2 (pJN24NDM) (Tables S4 and S5; Figure 3, Figures S3 and S4) [24,25,26,27]. The most prevalent plasmidic sequences were found in 16 strains and distributed into clusters A and C, which were similar to the IncX3 replicon-type *K. pneumoniae* pNDM-HN380 from China [24]. The *bla*_NDM-4_-encoding plasmid pEncl-922cz of the incompatibility group IncX3 from the Czech Republic has been published previously [11]. pEncl-922cz was identical to the respective sequences of *bla*_NDM-4_-encoding plasmids recovered in the same hospital during 2016 (such as strain Encl-44578 included in the present study) [12] but differed by the insertion of a Tn*3*-like transposon downstream of the *topB* gene compared with pNDM-HN380 and other IncX3 replicon types, such as the *bla*_NDM-5_-producing *K. pneumoniae* pNDM-MGR194 from India [28].

The *bla*_NDM_-carrying pKOX_NDM-1 strains (*n* = 8) were distributed into cluster A (Tables S3 and S5). The genome sequence and the *bla*_NDM-1_-harboring plasmid of strain P1 from Iran have been published previously [9]. *bla*_NDM-1_ was carried on a pKOX_NDM1-like plasmid, which is a non-transferable IncFII_Y_-type plasmid first reported in Taiwan [24,29], and later, in another *E. cloacae* complex, *K. pneumoniae*, *K. oxytoca* and *Serratia marcescens* isolates were recovered in Romania [30]. Different evolutionary events, including single-nucleotide-level change, indels and recombination events were observed among pKOX_NDM-1-like plasmids. The *bla*_NDM_-carrying pGUE-NDM strains (*n* = 3) were distributed into clusters A and B (Tables S3 and S5). The IncFII-type plasmid pGUE-NDM (IncFII) was first described in an *E. coli* MLST ST131 isolate from France [31] and plasmids from other Enterobacterales [32]. The EC-ML-559 strain from Greece (cluster C) carried a *bla*_NDM-1_-harboring structure (Tables S3 and S5) found in *Klebsiella pneumoniae* subsp. *pneumoniae* strain KPX plasmid pKPX-1 from Taiwan [15,33] and *Enterobacter hormaechei* subsp. *xiangfangensis* strain ST114 plasmid pLAU_ENM30_NDM1 from Lebanon [13]. Finally, *bla*_NDM-1_-harboring contigs of strain RIVM_C015180 from the Netherlands and strain E472 from Singapore were similar to plasmids pM214_AC2 (IncA/C) [26] and pJN24NDM (IncN2) [27], respectively (Tables S3 and S5), which have been previously described in *bla*_NDM_-harboring plasmids of *E. coli*.

The conjugative regions (*oriT*, relaxase gene, T4CP gene and T4SS gene cluster) of the self-transmissible MGEs were characterized for the ST182 strains that have caused the outbreaks in the Czech Republic [11,12] and Greece [14,15]. In the *bla*_NDM-1_-harboring strain EC-ML559 from Greece (Biosample SAMN33955250), all four conjugative regions were predicted: the *oriT* (region: 13,705–13,786), the relaxase gene (region: 14,142–16,070), the T4CP gene (region: 19,453–21,645) and the T4SS gene cluster (region: 19,453–44,454). In plasmid pEncl-922cz from the Czech Republic (Biosample SAMN08436979), no *oriT* region was predicted, but a relaxase gene (region 34,117–35,277), the gene encoding the type IV coupling protein (T4CP, region: 21,075–22,910) and the gene cluster for the bacterial type IV secretion system (T4SS, region: 20,284–33,022) were predicted. 

## 3. Discussion

The ECC mainly comprises six *Enterobacter* species (*E. asburiae, E. cloacae, E. hormaechei, E. kobei, E. ludwigii* and *E. nimipressuralis*); however, the accurate identification of species/subspecies of the genus *Enterobacter* by routine identification techniques, as well as 16S rRNA and housekeeping genes, has often been inconsistent [1,16]. Thus, reclassification of species and subspecies of the genus *Enterobacter* by phylogenetic studies based on whole-genome DNA-DNA hybridizations and sequencing is challenging and ongoing [1,16,34]. A global study of carbapenemase-producing ECC isolates collected during 2008–2014 revealed that the most common identified carbapenemase was VIM MBL, followed by NDM MBL, class A KPC, class D OXA-48 and IMP MBL [8]. As observed with other carbapenemase-producing ECC strains, *bla*_NDM_-producing ECC strains were also found to mainly belong to four STs, named ST114, ST93, ST90 and ST78. In the present assay, we performed phylogenetic analysis for 646 STs identified among all 4685 *E. hormaechei* WGS assemblies deposited in public databases, which revealed that ST182 is an emerging lineage in ECC strains. ECC ST182 strains were predicted in silico to harbor plasmids commonly found among multidrug-resistant bacteria, which have acquired antimicrobial resistance and virulence genes, whereas different *bla*_NDM_-harboring plasmid types among ST182 ECC strains were distributed in all sublineages.

It has been suggested that global travel has facilitated the rapid spread of NDM from its initial emergence in India [5] to all continents since the importation of NDM producers has been associated with patients having a history of travel [3,4,5,6,7,8,13,35,36,37]. A recent study from Israel has shown that most of the *bla*_NDM_-harboring Enterobacterales possessed nine different MGE modules, variably distributed across species and hospitals [35]. In another study, the role of mobile genetic elements in the global dissemination of the *bla*_NDM_ was investigated and it was estimated that *bla*_NDM_ emerged on a Tn*125* transposon before 1985 but only reached global prevalence around a decade after its first recorded observation in 2008 [36]. The global dissemination of the *bla*_NDM_ gene was primarily driven by successive between-plasmid transposon jumps [36]. In *K. pneumoniae*, different trajectories have been shown for the spread of carbapenemase genes, including via one plasmid/multiple lineages (*bla*_OXA-48_-like), multiple plasmids/multiple lineages (*bla*_VIM_, *bla*_NDM_) and multiple plasmids/one lineage (*bla*_KPC_) [37]. The findings of the current study revealed that *E. hormeacei* ST182 WGS assemblies deposited in public databases were collected from 2002 to 2021, and during this period, we have identified both WGS assemblies carrying *bla*_NDM_ and WGS assemblies with no *bla*_NDM_ genes. No clustering over time was observed for the two groups of strains or the different *bla*_NDM_ plasmid types, suggesting that strains without *bla*_NDM_ genes have been distributed globally, and then *bla*_NMD_ genes were diffused in different genomic clusters.

In a previous study, a common *bla*_NDM_ genetic structure on plasmid pNDM-U.S. was identified in 14 different ECC clones obtained from six countries spanning four continents [6]. Moreover, in some cases, certain mobile genetic elements with carbapenemase genes were found associated with the geographic distribution of clades, clones and species, suggesting that these mobile elements have the ability to move between clones and clades of ECC on a global scale. Several surveys have shown that the *bla*_NDM_ genes were distributed across a large number of STs in the most prevalent species of Enterobacterales (*E. coli, K. pneumoniae* and *Enterobacter* spp.), with no predominant lineages, suggesting that there are no obvious high-risk clones of *bla*_NDM_-producing strains [3,4,7]. In the current study, *bla*_NDM_-harboring contigs showed similarities with six different plasmid types [. The most prevalent IncX3 replicon-type pNDM-HN380-like structures were found in four continents (Asia, Europe, North America and Africa) and diffused into genomic clusters (sublineages) A and C. Similarly, the three different IncFII-type genetic structures were also distributed into different continents and/or genomic clusters: the pKOX_NDM-1 in Asia, Europe, North and South America (cluster A), the pGUE-NDM-like structures in Asia (clusters A and B) and the pKPX-1 in Europe (cluster C). Thus, different *bla*_NDM_-carrying plasmids were diffused among strains of the same genomic cluster (sublineage), and on the other hand, the same *bla*_NDM_-carrying plasmid could be found in strains belonging to different sublineages of ST182. Therefore, no associations were observed between the genetic clusters and the country/continent of isolation, the presence of the *bla*_NDM_ alleles and the plasmid types.

Finally, in the present survey, we have performed a phylogenomic analysis and in silico prediction of antimicrobial resistance genes, virulence genes and plasmid types of *E. hormaechei* ST182 WGS assemblies deposited in public repositories. It should be noted that there are some limitations considering this approach. Firstly, phenotype testing or functional studies are required to determine whether some of the detected genes could confer resistance. Secondly, the existing databases for virulence factors (e.g., Virulence Factor Database—VFDB; available at http://www.mgc.ac.cn/VFs/, accessed on 15 January 2024) do not include data for *Enterobacter* species. Therefore, there may be additional virulence genes that were not predicted in this survey. Thirdly, plasmid reconstruction was not performed due to short sequencing reads. It should be noted that plasmids are difficult to reconstruct from WGS data. NGS assembly programs tend to return short contigs of heterogeneous origin. On the other hand, alignment-based tools tend to miss diverged plasmids, while learning-based tools often have lower precision. In some studies, the combination of short and long sequencing read WGS strategies has been used []. Another limitation of the present descriptive survey is that it included only sequenced ECC isolates in the NCBI and PubMLST public repositories, which are deposited randomly by users, and there may be a bias towards multidrug-resistant strains; thus, they do not represent the global molecular epidemiology of ECC isolates. Further epidemiological and molecular surveillance studies at a global scale would define the prevalence of the ST182 lineage in ECC strains.

## 4. Materials and Methods

### 4.1. Bacterial Isolates, Genome Sequences and Phylogenetic Analysis

A total of 4685 WGS assemblies of *E. hormaechei* isolates with available MLST profiles were analyzed. We have retrieved WGS assemblies from the Pathogenwatch database [38] and the PubMLST *Enterobacter cloacae* database (available at: https://pubmlst.org/organisms/enterobacter-cloacae; accessed on 15 January 2024) [39], which include WGS assemblies from public repositories, such as the European Nucleotide Archive (ENA) and NCBI. Additionally, we have searched the PubMLST database for the presence of the alleles of ST182 (*dnaA*-49, *fusA*-20, *gyrB*-19, *leuS*-44, *pyrG*-90, *rplB*-24, *rpoB*-32) in other MLST profiles, which were found in 62, 174, 89, 220, 9, 27 and 125 MLST profiles (STs), respectively. Since alleles *pyrG*-90 and *rplB*-24 are present in fewer MLST profiles (9 and 27 profiles, respectively) compared with the other MLST alleles of ST182, we have also searched the NCBI database for these alleles (*pyrG*-90 and *rplB*-24), and the MLST 2.0 tool (available at: https://cge.food.dtu.dk/services/MLST/ (accessed on accessed on 15 January 2024), Center for Genomic Epidemiology, Technical University of Denmark) was used to define the MLST STs so as to retrieve any additional WGS assemblies of ST182. In the final dataset, a total of 55 WGS assemblies of ST182 were included. The genetic relationships and groups of STS were formed by linking all STs that were single-locus variants (SLVs), known as clonal complexes (CCs), by using the goeBURST and the PHYLOViZ version 2.0 software (available at http://www.phyloviz.net/, accessed on 15 January 2024) [40].

The phylogenomic analysis of the WGS assemblies of ST182 strains was performed using the Reference sequence Alignment based Phylogeny (REALPHY) tool (available at: https://realphy.unibas.ch/realphy/, accessed on 15 January 2024) [41]. The WGS assembly of the type strain *E.cloacae subsp. cloacae* NCTC9394 (GenBank accession no. FP929040.1) was used as a reference sequence. Of note, the taxonomic classification of the type strain NCTC9394 was updated on 04/08/2020 from *Enterobacter cloacae* to *Enterobacter hormaechei* (https://www.ncbi.nlm.nih.gov/nuccore/NC_021046.1?report=genbank, accessed on 15 January 2024). Single-nucleotide polymorphisms (SNPs) were extracted using the Galaxy Server (available at https://usegalaxy.org/, accessed on 15 January 2024) from the aligned set of orthologous sites obtained by REALPHY [42], and phylogenetic analysis was performed using the MEGA version 11 software [19]. Visualization of the timeline of the isolation, the geographical distribution and the phylogenetic tree of the 55 ST182 ECC strains were performed using the Microreact web application (available at: https://microreact.org/, accessed on 15 January 2024) [43].

### 4.2. Identification of MGEs, Antimicrobial Resistance Genes and Virulence Factors and Plasmid Analysis

BLASTN (available at: https://blast.ncbi.nlm.nih.gov/Blast.cgi, accessed on 15 January 2024), the KmerResistance 2.2 tool and the Mobile Element Finder tool (available at: https://cge.food.dtu.dk/services/; accessed on 25 February 2024) were used to query the sequence assemblies for identification of the *bla*_NDM_-harboring contigs, MGEs, plasmids and their relation to antimicrobial resistance genes and virulence factors on the genomes [44,45]. k-mer alignment examines the co-occurrence of k-mers between the WGS data and a database of resistance genes and scales well for large redundant databases [21]. The GC content of the WGS assemblies was calculated by using the GC-profile (available at: http://tubic.tju.edu.cn/GC-Profile/, accessed on 15 January 2024) [46] and the GCdraw (available at: http://www.endmemo.com/bio/gcdraw.php, accessed on 15 January 2024) online tools. The *bla*_NDM_-harboring contigs of the isolates were analyzed with the oriTfinder tool [47] so as to explore the presence of conjugative regions of the self-transmissible MGEs: the origin of transfer site (*oriT*), the relaxase gene, the gene encoding the type IV coupling protein (T4CP) and the gene cluster for bacterial type IV secretion system (T4SS). A conjugative plasmid must possess all the conjugative regions, whereas a transmissible plasmid must possess at a minimum an *oriT* and usually a relaxase, but this can be provided *in trans* [47].. BlastN comparisons of the *bla*_NDM_-harboring contigs with plasmid sequences retrieved from the NCBI were performed using the BLAST Ring Image Generator (BRIG) version 0.95 software (available at: https://brig.sourceforge.net/, accessed on 15 January 2024) [48].

## 5. Conclusions

In the present survey, we have shown via phylogenetic analysis that the multidrug-resistant ST182 is an emerging lineage in ECC strains, representing a distinct clonal complex among *bla*_NDM_-carrying ECC strains. *E. hormarchei* ST182 strains retrieved from public databases were distributed into three genomic clusters (sublineages), which contained strains recovered from five different continents. Both *bla*_NDM_-carrying ST182 strains and strains with no *bla*_NDM_ were diffused into the three ST182 sublineages. Moreover, different plasmid types have been spread among the three genetic clusters of ST182, whereas no associations were observed between the genetic clusters and plasmid types. The diversity of the *bla*_NDM_-harboring genetic structures identified among ST182 isolates denotes different routes of *bla*_NDM_ acquisition into the ST182 clusters worldwide. These findings suggest that ST182 strains without *bla*_NDM_ genes emerged and spread initially and later on acquired the *bla*_NDM_ genetic structures via horizontal gene transfer from other bacteria in the recent past. Furthermore, ST182 has already caused outbreaks in the Czech Republic and Greece, and therefore, it has the potential to cause outbreaks worldwide. Vigilance and continuous molecular-typing-based surveillance seem mandatory among ECC strains in order to understand the further expansion of the emerged *E. hormarchei* ST182 and restrain its dissemination.

## Figures and Tables

**Table 1:** Distribution of 55 ST182 *E. hormaechei* isolates into genomic clusters (sublineages), continent/country of isolation and *bla*_NDM_ variants.

Genomic Clusters	Continent/Country of Isolation	*bla*_NDM_ Variants					Total
		*bla*_NDM-1_	*bla*_NDM-4_	*bla*_NDM-5_	*bla*_NDM-7_	None	
Cluster A (No. of isolates)		19	2	1	2	13	37
	Africa			1		3	4
	Senegal					2	2
	Togo			1			1
	Tunisia					1	1
	Asia	10				1	11
	China	2				1	3
	Iran	1					1
	Myanmar	3					3
	Pakistan	2					2
	Philippines	1					1
	Singapore	1					1
	Europe	4	2		1	5	12
	Czech Republic		2				2
	Germany					1	1
	Netherlands	3			1		4
	Spain					3	3
	United Kingdom	1				1	2
	North America	4			1	4	9
	Guatemala	1					1
	USA	3			1	4	8
	South America	1					1
	Mexico	1					1
Cluster B (No. of isolates)		1				9	10
	Asia	1				1	2
	India	1					1
	Singapore					1	1
	Europe					4	4
	Netherlands					1	1
	Switzerland					1	1
	United Kingdom					2	2
	North America					2	2
	USA					2	2
	South America					2	2
	Colombia					2	2
Cluster C (No. of isolates)		4		1		3	8
	Asia	3				1	4
	China	1					1
	Singapore	1					1
	Thailand					1	1
	Viet Nam	1					1
	Europe	1					1
	Greece	1					1
	North America			1		2	3
	USA			1		2	3
	Total	24	2	2	2	25	55

## Data Availability

Not applicable.

## Supplementary Materials

The following supporting information can be downloaded at https://www.mdpi.com/article/10.3390/antibiotics13060535/s1, Figure S1: Evolutionary relationships of ST182 *E.hormaechei* WGS assemblies.; Figure S2: Genomic clusters, *bla*NDM variants, country, continent and year of isolation of 55 ST182 *E. hormaechei* WGS assemblies; Figure S3: Yearly distribution of ST182 *E. hormaechei* (a) *bla*_NDM_ variants and (b) plasmid types of *bla*_NDM_-carrying WGS assemblies during 2011–2021; Figure S4: BlastN comparisons of the nucleotide sequences of *bla*_NDM_-harboring contigs; Table S1: Distribution of 646 MLST STs (n=4685 ECC isolates) into clonal complexes and singletons obtained with goeBURST analysis. Table S2: Characteristics of 55 ST182 *E. hormaechei* WGS assemblies; Table S3: Characteristics of 30 ST182 *E. hormaechei bla*_NDM_-carrying WGS assemblies; Table S4: In silico prediction of contig Inc types, antimicrobial resistance and virulence genes in 30 *bla*_NDM_-harboring ST182 *E. hormaechei* WGS assemblies. Table S5: Distribution of the 55 ST182 *E. hormaechei* isolates into genomic clusters, continent and country of isolation, *bla*_NDM_ variants and *bla*_NDM_-carrying plasmid types. References [42,49,50,51,52] are cited in the Supplementary Materials.
