# Supplementary material for: Comparative Genomics of an Emerging Multidrug-Resistant blaNDM-Carrying ST182 Lineage in Enterobacter cloacae Complex
Source: Antibiotics (Basel). 2024 Jun 8;13(6):535. doi: 10.3390/antibiotics13060535 (PMC11200890; doi:10.3390/antibiotics13060535)
Supplement: Supplementary file 1 [file antibiotics-13-00535-s001.zip › antibiotics-3037667-supplementary.pdf]

Supplementary Table S1. Distribution of 646 MLST STs (n=4685 ECC isolates) into clonal complexes and singletons obtained with goeBURST analysis.

**Statistics**	**Population**	**Clonal groups (CCs)**	**Singletons**
STs	646	400	246
isolates	4685	3953	732
groups	320	74	246
edges	441 [441 0 0]	441 [441 0 0]	0 [0 0 0]
forest edges	326 [326 0 0]	326 [326 0 0]	0 [0 0 0]
edges without ties	191	191	0
tiebreaks by SLV	128	128	0
tiebreaks by DLV	4	4	0
tiebreaks by TLV	0	0	0
tiebreaks by FRQ	3	3	0
tiebreaks by ID	0	0	0

Supplementary Table S2. Characteristics of 55 ST182 *E. hormaechei* WGS assemblies.

**BIOSAMPLE**	**STRAIN ALIAS**	**CONTINENT**	**COUNTRY**	**YEAR**	***bla*NDMvariant**	**CLUSTER**
SAMD00099782	M515	Asia	Myanmar	2016	*bla*$_{NDM-1}$	A
SAMD00194914	MY196	Asia	Myanmar	2016	*bla*$_{NDM-1}$	A
SAMD00431577	SAMD00431577	Asia	Myanmar	2016	*bla*$_{NDM-1}$	A
SAMEA6368456	RIVM_C015180	Europe	Netherlands	2017	*bla*$_{NDM-1}$	A
SAMEA7707283	RIVM_C019172	Europe	Netherlands	2019	*bla*$_{NDM-1}$	A
SAMEA7707293	RIVM_C019489	Europe	Netherlands	2019	*bla*$_{NDM-1}$	A
SAMEA8581547	PP301	Asia	Pakistan	2016	*bla*$_{NDM-1}$	A
SAMEA8581614	PS171	Asia	Pakistan	2016	*bla*$_{NDM-1}$	A
SAMN04431002	GCID_ENTES_00096;SMART_1342	North America	Guatemala	2014	*bla*$_{NDM-1}$	A
SAMN07355852	HBY	Asia	China	2017	*bla*$_{NDM-1}$	A
SAMN07501531	AZ_664	Asia	Philippines	2013	*bla*$_{NDM-1}$	A
SAMN08932725	SCEH020080	Asia	China	2016	*bla*$_{NDM-1}$	A
SAMN10252240	Sentry-2017-1013030	South America	Mexico	2017	*bla*$_{NDM-1}$	A
SAMN22068468	2021QW-00060	North America	USA	2021	*bla*$_{NDM-1}$	A
SAMN22377001	P1	Asia	Iran	2012	*bla*$_{NDM-1}$	A
SAMN24297073	222410	Europe	UK	2016	*bla*$_{NDM-1}$	A
SAMN24700281	EC1268	Asia	Singapore	2015	*bla*$_{NDM-1}$	A
SAMN25161196	2021QW-00088	North America	USA	2021	*bla*$_{NDM-1}$	A
SAMN25161198	2021QW-00093	North America	USA	2021	*bla*$_{NDM-1}$	A
SAMN32638338	PEER 1096	Asia	India	2021	*bla*$_{NDM-1}$	B
SAMN04252918	GCID_ENTES_00038;SMART_598	Asia	Viet Nam	2011	*bla*$_{NDM-1}$	C
SAMN15904743	hkcpe74	Asia	Hong Kong	2012	*bla*$_{NDM-1}$	C
SAMN21590478	E472	Asia	Singapore	Uknown	*bla*$_{NDM-1}$	C
SAMN33955250	EC-ML559	Europe	Greece	2021	*bla*$_{NDM-1}$	C
SAMN08436979	Encl-922	Europe	Czech Republic	2012	*bla*$_{NDM-4}$	A
SAMN08450112	Encl-44578	Europe	Czech Republic	2016	*bla*$_{NDM-4}$	A
SAMN07503715	ECLO_616294_SO	Africa	Togo	2016	*bla*$_{NDM-5}$	A
SAMN14669245	2019GO-00055	North America	USA	2019	*bla*$_{NDM-5}$	C
SAMEA7707309	RIVM_C029661	Europe	Netherlands	2020	*bla*$_{NDM-7}$	A
SAMN22573158	2021GN-00156	North America	USA	2021	*bla*$_{NDM-7}$	A
SAMEA7071344	H163100552	Europe	UK	2016	None	A
SAMEA7962787	AI2760	Europe	Spain	2018	None	A
SAMEA8065826	AI2747	Europe	Spain	2018	None	A
SAMN03283617	GCID_ENTE_00053	North America	USA	2012	None	A
SAMN03283626	GCID_ENTE_00049	North America	USA	2012	None	A
SAMN03283632	GCID_ENTE_00066	North America	USA	2012	None	A
SAMN04252905	GCID_ENTES_00025; SMART_409	Europe	Spain	2011	None	A
SAMN04431017	GCID_ENTES_00111;SMART_1169	Africa	Tunisia	2014	None	A
SAMN14516611	And2481	Europe	Germany	2013	None	A
SAMN16558068	1800086	North America	USA	2016	None	A
SAMN33748146	EC84	Asia	China	2015	None	A
SAMN35822989	Eh202_LBHALD	Africa	Senegal	2018	None	A
SAMN36341330	Eh8322_LBHALD	Africa	Senegal	2018	None	A
SAMEA2273150	MDR0039-sc-1791683	Europe	UK	2002	None	B
SAMEA2273329	MDR0244-sc-1791881	Europe	UK	2006	None	B
SAMN03277204	CIDEIMsCOL17	South America	Colombia	2012	None	B
SAMEA4427188	SCP26-50	Europe	Netherlands	Uknown	None	B
SAMN03283690	GCID_ENTE_00121; 44527	South America	Colombia	2014	None	B
SAMN18207028	ENCL_547	North America	USA	2018	None	B
SAMN20064946	N1730	Europe	Switzerland	2020	None	B
SAMN20967435	E929	Asia	Singapore	Uknown	None	B
SAMN07610229	UM_CRE_44	North America	USA	2015	None	B
SAMN25226637	2021DK-00183	North America	USA	2021	None	C
SAMN25226704	2021DK-00272	North America	USA	2021	None	C
SAMN32262091	CUVET18-121; Ecl1	Asia	Thailand	2018	None	C

Supplementary Table S3. Characteristics of 30 ST182 *E. hormaechei* *bla*$_{NDM}$-carrying WGS assemblies.

Biosample	Strain	Accession No; (size, %GC content)	*bla*NDM-harboring contig (size)	*bla*$_{NDM}$ variant
**pNDM-HN380; X104760 (IncX3)**				
SAMN07355852	HBY	NMVR01000000.1 (5,790,239 bp; 52.9%)	NMVR01000120.1; (3,335bp)	*bla*$_{NDM-1}$
SAMN04431002	SMART_1342	LRJK01000000.1 (4,909,094 bp; 55.3%)	NZ_LRJK01000027.1 (13,396bp)	*bla*$_{NDM-1}$
SAMN04252918	SMART_598	LPPQ01000000.1 (5,090,561 bp; 55.1%)	LPPQ01000025 (11,228bp)	*bla*$_{NDM-1}$
SAMN07501531	AZ 664	DACOIW010000000 (5,171,314 bp, 55.1%)	1. DACOIW010000076 (9,086bp) 2. DACOIW010000145 (8,220 bp)	*bla*$_{NDM-1}$
SAMN07503715	ECLO_616294_SO	NRJJ00000000.1 (4,822,414bp; 53.21%)	NZ_NRJJ01000307.1 (5,630bp)	*bla*$_{NDM-1}$
SAMD00099782	M515	PDT000431889_1 (5,209,379 bp; 54.88%)	1. DACOQQ010000041.1 (8,852bp) 2. DACOQQ010000093.1 (6,231bp)	*bla*$_{NDM-1}$
SAMD00194914	MY196	PDT001202126 (5,023,457 bp; 54.93%)	1. DAFILT010000050.1 (8,948bp) 2. DAFILT010000085.1 (1,539bp)	*bla*$_{NDM-1}$
SAMN08932725	SCEH020080	SRR7026274 (5,116,489bp; 54.9%)	SAMN08932725_NODE_32 (8,106bp)	*bla*$_{NDM-1}$
SAMN15904743	hkcpe74	SRR12517442 (5,184,472bp; 54.7%)	SAMN15904743_NODE_222 (1,237bp)	*bla*$_{NDM-1}$
SAMEA7707293	RIVM_C019489	ERR4977540 (5065033bp; 55.3%)	SAMEA7707293_NODE_34 (13,230bp)	*bla*$_{NDM-1}$
SAMD00431577	SAMD00431577	DRR334946 (4,992692bp; 54.9%)	SAMD00431577_NODE_48 (9,044bp)	*bla*$_{NDM-1}$
SAMN08436979	Encl-922	NZ_PQXG01000000.1 (4,884,183; 56.27%)	MG252892 (53,683bp)	*bla*$_{NDM-4}$
SAMN08450112	Encl-44578	NZ_QBPF01000000.1 (4,832,725; 53.35%	NZ_QBPF01000054.1 (5,798bp)	*bla*$_{NDM-4}$
SAMN14669245	2019GO-00055	SRR11594612 (4,868,786bp; 55.3%)	SAMN14669245_NODE_19 (4,6873bp)	*bla*$_{NDM-5}$
SAMN22573158	2021GN-00156	SRR16609045 (4,920,998bp; 55.2%)	SAMN22573158_NODE_34 (5,661bp)	*bla*$_{NDM-7}$
SAMEA7707309	RIVM_C029661	ERR4977554 (5,060,221bp; 55.1%)	SAMEA7707309_NODE_43 (5,615bp)	*bla*$_{NDM-7}$
**pKOX_NDM-1; NC_021501.1 (IncFII)**				
SAMN22377001	P1	JAJCVN000000000.1 (5,015,83bp; 52.54%)	NZ_JAJCVN010000041.1 (18,792bp)	*bla*$_{NDM-1}$
SAMN24297073	222410	SRR17308905 (5,001,248bp; 55.2%)	SAMN24297073_NODE_45 (19,412bp)	*bla*$_{NDM-1}$
SAMN24700281	EC1268	SRR17519753 (5,016,482bp; 55.3%)	SAMN24700281_NODE_31 (15,288bp)	*bla*$_{NDM-1}$
SAMN25161196	2021QW-00088	SRR17696410 (5,132,726bp; 55.1%)	SAMN25161196_NODE_28 (21,092bp)	*bla*$_{NDM-1}$
SAMN25161198	2021QW-00093	SRR17696408 (5,142,242bp; 55.1%)	SAMN25161198_NODE_26 (21,092bp)	*bla*$_{NDM-1}$
SAMEA7707283	RIVM_C019172	ERR4977532 (5,006,535bp;55.2%)	SAMEA7707283_NODE_34 (17,894bp)	*bla*$_{NDM-1}$

SAMN10252240	Sentry-2017-1013030	SRR8306302 (4,988,526bp; 55.2%)	SAMN10252240_NODE_24 (77,041bp)	*bla*$_{NDM-1}$
SAMN22068468	2021QW-00060	SRR16212278 (5,033,415bp; 55.2%)	SAMN22068468_NODE_26 (19,874bp)	*bla*$_{NDM-1}$
**pGUE-NDM; JQ364967.1 (IncFII)**				
SAMN32638338	PEER1096	PubMLST id: 1351; (4,713,253 bp; 55.35%)	Peer1096spades_contig_16 (Length: 9918bp, 56.9%)	*bla*$_{NDM-1}$
SAMEA8581547	PP301	ERR5751977 (5,108,989bp; 55.0%)	SAMEA8581547_NODE_52_l(72,96 bp)	*bla*$_{NDM-1}$
SAMEA8581614	PS171	ERR5751910 (5041435bp; 55.2%)	SAMEA8581614_NODE_28 (16,238bp)	*bla*$_{NDM-1}$
**pKPX-1 AP012055.1 (IncFII)**				
SAMN33955250	EC-ML559	PubMLST id: 1241; JARUPS000000000.1 (4,958,007 bp; 55.07%)	JARUPS010000023.1 (61,729bp; 53.7%)	*bla*$_{NDM-1}$
**pM214_AC2; AP018143.1(IncA/C)**				
SAMEA6368456	RIVM_C015180	ERR3712849 (5,004,082bp; 55.2%)	SAMEA6368456_NODE_54 (7,959bp)	*bla*$_{NDM-1}$
**pJN24NDM1; MK368725.1 (IncN2)**				
SAMN21590478	E472	SRR16079366 (5,176,427bp;54.8%)	SAMN21590478_NODE_37 (20,112bp)	*bla*$_{NDM-1}$

Supplementary Table S4. *In silico* prediction of contig Inc types, antimicrobial resistance and virulence genes in 30 *bla*$_{NDM}$-harbouring ST182 *E. hormaechei*WGS assemblies.

Biosample accession	Genomic Cluster	Contig Inc types	Antimicrobial resistance genes	Virulence genes
SAMD00099782	A	IncHI2,IncHI2A,IncR, IncFII(K)/repB(R1701)	**blaACT-16, blaNDM-1,blaTEM-1, blaSFO-1, blaNDM-1, blaCTX-M-15,** *aph(6)-Id, aac(3)-IId, aadA, aadA16, aph(3')-Ia, aac(6')-Ib-cr, aph(3'')-Ib, mcr-9*	*nlpI, terC, traT*
SAMD00194914	A	IncHI2,IncHI2A,IncR, IncFII(K),repB(R1701)	**blaACT-16, blaNDM-1,blaTEM-1, blaNDM-1,** *aph(6)-Id,aadA1, aph(3'')-Ib,mcr-9, formA,qacE,OqxA,OqxB, fosA,catA2,sul1,dfrA12*	*nlpI, terC, traT*
SAMD00431577	A	IncHI2,IncHI2A,IncR, repB(R1701)	**blaACT-16, ,blaNDM-1,blaTEM-1,blaNDM-1,***aph(6)-Id,aadA1,aph(3'')-Ib,mcr-9,formA qacE,OqxA, OqxB,fosA,catA2,sul1*	*nlpI, terC, traT*
SAMEA6368456	A	IncFIB(pECLA)/IncFII(pECLA),IncFII,IncR Col(pHAD28)	**blaACT-16, blaNDM-1,blaTEM-1,** *aph(6)-Id aadA1, aph(3'')-Ib,mcr-9, formA, qacE OqxA, OqxB,fosA, catA2,sul1,dfrA12*	*nlpI, terC, traT*
SAMEA7707283	A	IncFIB(pECLA)/IncFII(pECLA), IncFII(Yp),Col(pHAD28),	**blaACT-16, blaNDM-1, blaOXA-1,blaTEM-1, blaCTX-M-15** *,aph(6)-Id, rmtC, aac(6')-Ib-cr, aph(3'')-Ib,aac(3)-Iia,OqxA, OqxB, fosA, mph(A) ,catB3 ,qnrB1 sul1,sul2, tet(D), dfrA14*	*nlpI, terC*
SAMEA7707293	A	IncFIB(pECLA)/IncFII(pECLA),IncFIA(pBK30683), IncM1,IncR, repB(R1701)	**blaACT-16,blaNDM-1,blaOXA-1,blaOXA-48,blaTEM-1,blaGES-5,blaLAP-2, blaCTX-M-15,***aph(6)-Id,aph(3'')-Ib,aph(3'')-Ib,aac(6')-Ib,aadA1,aph(3')-VIb,aac(3)-IIa,qacE,OqxA,OqxB, fosA,catB3,qnrS1,sul1,sul2,tet(A),dfrA1,dfrA14*	*nlpI, terC*
SAMEA8581547	A	IncFIB (pECLA),IncFIB (pHCM2),IncFIA(pBK30683), IncM1(pKPC_NDM3594),ColR NAI	**blaACT-16,blaNDM-1,blaOXA-1,blaOXA-10,***aadA1,aac(6')-Ib-cr,qacE,OqxA,OqxB,fosA,catB3,ARR-3,sul1,dfrA27,dfrA14*	*nlpI, terC,shiB*
SAMEA8581614	A	IncFIB(pECLA)/IncFII(pECLA),IncFIB(pHCM2), IncFII(Yp), IncFIB(pB171)	**blaACT-16,blaNDM-1,blaOXA-1, blaTEM-1,blaCTX-M-15,** *aph(6)-Id,rmtC,aac(6')-Ib-cr,aph(3'')-Ib,aac(3)IIa, fosA,catB3, OqxA,OqxB,qnrB1,sul1,sul2,tet(A),tet(D),dfrA14*	*nlpI, terC*
SAMN04431002	A	IncFIB(pECLA),IncFII(pECLA) ,IncX3	**blaACT-16, blaNDM-1, blaTEM-1B,blaCTX-M-15, blaSHV-12,***aadA2b, aph(6)-Id, aph(3'')-Ib, fosA, ant(2'')-Ia, qacE, sul1, sul2, qnrA1,dfrA14,*	*nlpI, terC*
SAMN07355852	A	IncFIB(pHCM2), IncFII(Yp), IncHI2A, IncHI2, IncX3	**blaACT-16, blaNDM-1,blaTEM-1,blaCTX-M-3, blaDHA-1, blaKPC-2,***msr(E), aadA1, aac(6')-Ib-cr, armA, qacE, fosA, sul1, qnrB4, mcr-9*	*nlpI, terC*
SAMN07501531	A	IncFII(pECLA)/IncFIB(pECLA), IncX3, IncN	**blaACT-16,blaNDM-1,blaCTX-M-15,blaOXA-1, blaTEM-1, blaSHV-12,***aac(3)-IIa, aac(6')-Ib-cr, aph(3'')-Ib, aph(6)-Id, tet(A), dfrA14, fosA, catB3, sul2, OqxA, OqxB, qnrB1, qnrS1*	*nlpI, terC*
SAMN08932725	A	IncFIB(pB171),IncFII(Yp),IncH I2,IncHI2A,IncX3,Col(pHAD2 8)	**blaACT-16,blaNDM-1,blaCTX-M-9,blaTEM-1,blaSHV-12,***aph(6)-Id,ant(2'')-Ia,aac(3)-IId,aadA2,aph(3'')-Ib,qacE,OqxA,OqxB, fosA,mph(A),qnrA1,sul1,sul2,tet(A),dfrA12*	*nlpI, terC*
SAMN10252240	A	IncFIB(pECLA)/IncFII(pECLA),IncFII(Yp), IncFIB(pB171)	**blaACT-16,blaNDM-1, blaOXA-1,blaTEM-104,blaCTX-M-15,***aph(3')-VI,aph(6)-Id,rmtC,aac(6')-Ib-cr,aph(3'')-Ib,formA ,OqxA,OqxB,fosA,catB3,floR,qnrB1, sul1, sul2, tet(A), dfrA14*	*nlpI, terC*
SAMN22068468	A	IncFIB(pECLA)/IncFII(pECLA),IncFII(Yp),IncFIB(pB171), Col(pHAD28)	**blaACT-16,blaNDM-1,blaOXA -1,** *aac(6')-Ib-cr,aac(3)-IIa,rmtC,OqxA,OqxB,fosA,catB3,qnrB1sul1,tet(A),dfrA14*	*nlpI, terC*
SAMN22377001	A	IncFII(pECLA)/IncFIB(pECLA), IncFII(Yp), IncFIB(pB171)	**blaACT-16, blaNDM-1, blaCTX-M-15,blaOXA-1, blaTEM-1,** *aph(6)-Id, aac(6')-Ib-cr, aph(3'')-Ib,aac(3)-IIa, OqxA, OqxB, rmtC, fosA, catB3, tet(D), sul1, sul2, dfrA14, qnrB1*	*nlpI, terC*
SAMN24297073	A	IncFIB(pECLA)/IncFII(pECLA),IncFII(Yp), IncFIB(pB171), Col440I	**blaACT-16,blaNDM-1,blaOXA-1,blaTEM-1,blaCTX-M-15,** *aph(6)-Id,rmtC,aac(6')-Ib-cr_1,aph(3'')-Ib,aac(3)-IIa, OqxA,OqxB,fosA,catB3,qnrB1,sul1,sul2,tet(A),tet(D),dfrA14*	*nlpI, terC*
SAMN24700281	A	IncFIB(pECLA)/IncFII(pECLA),IncFII(Yp),IncFIB(pB171), Col440I	**blaACT-16,blaNDM-1,blaOXA-1,blaTEM-1,blaCTX-M-15,***aph(6)-Id,aadA2,rmtC,aac(6'),aph(3'')-Ib,aac(3)-IIa,qacE_1,OqxA,OqxB,fosA,mph(A),catB3,qnrB1,sul1,sul2,tet(A),tet(D),dfrA12,dfrA14*	*nlpI, terC*

SAMN25161196	A	IncFIB(pECLA)/IncFII(pECLA),IncFII(Yp),IncFIA(pBK30683),IncFIB(pB171),IncN,IncR,Col(pHAD28)	**blaACT-16,blaNDM-1,blaOXA-1,blaTEM-1,blaCTX-M-3,***aac(6′)-Ib3,rmtC,aac(3)-IIa,OqxA,OqxB,fosA,catB3,ARR-3,sul1,dfrA14*	*nlpI, terC, mrkA, astA*
SAMN25161198	A	IncFIB(pECLA)/IncFII(pECLA),IncFIA(pBK30683),IncFII(Yp),IncFIB(pB171),IncN,IncR,Col(pHAD28)	**blaACT-16,blaNDM-1,blaOXA-1,blaTEM-1,blaCTX-M-3,***aac(3)-IIa, aac(6′)-Ib3,rmtC,OqxA,OqxB,fosA,catB3,qnrB1,ARR-3,sul1,tet(A),dfrA14,*	*nlpI, terC, mrkA, astA*
SAMN08436979	A	IncFII(pECLA)/IncFIB(pECLA), IncX3	**blaACT-16, blaNDM-4, blaOXA-1,blaTEM-1, blaCTX-M-15** *aac(3)-IIa, aac(6′)-Ib-cr, aph(6)-Id, aph(3′′)-Ib, fosA, catB3, sul2, tet(A), dfrA14, OqxA, OqxB, qnrB1*	*nlpI, terC*
SAMN08450112	A	IncFII(pECLA)/IncFIB(pECLA), IncX3	**blaACT-16, blaNDM-4, blaOXA-1,blaTEM-1, blaCTX-M-15** *aac(6′)-Ib-cr, aph(3′′)-Ib, aac(3)-IIa, aph(6)-Id, tet(A),fosA, dfrA14, catB3, sul2, qnrB1*	*nlpI, terC*
SAMN07503715	A	IncFIB(pECLA)/IncFII(pECLA), IncX3	**blaACT-16, blaNDM-5, blaTEM-1,***aph(6)-Id, aph(3′′)-Ib, aac(6′)-Ib-cr, aac(3)-IIa, catB3, tet(D), tet(A), dfrA14, fosA, sul2,qnrB1*	*nlpI, terC*
SAMEA7707309	A	IncFIB(pECLA)/IncFII(pECLA),IncFII(Yp),IncX3,Col(pHAD28)	**blaACT-16,blaNDM-7,blaOXA-1,blaTEM-1,blaCTX-M-15,***aph(6)-Id,aadA2,armA,aac(6′)-Ib-cr,aph(3′′)-Ib,aac(3)-IIa,qacE, OqxA,OqxB,fosA,msr(E),mph(E),catB3,qnrB1,sul1,sul2,tet(D) dfrA12,dfrA14*	*nlpI, terC*
SAMN22573158	A	IncFIB(pECLA)/IncFII(pECLA),IncX3	**blaACT-16,blaNDM-7,blaOXA-1,blaTEM-1,blaCTX-M-15**, *aph(6)-Id,aac(6′)-Ib-cr,aph(3′′)-Ib,aac(3)-IIa,OqxA_1,OqxB,fosA,catB3,qnrB1,sul2,tet(A),tet(D),dfrA14*	*nlpI, terC*
SAMN32638338	B	IncFIB(K),Col440I, Col440II	**blaACT-16, blaNDM-1, blaCTX-M-15,***aac(6′)-Ib,aadA1, aadA2,OqxA,OqxB, fosA, qacE, sul1, dfrA12,qnrS1*	*nlpI*
SAMN04252918	C	IncFIB(pECLA)/IncFII(pECLA),Col(pHAD28),IncFII(Yp)	**blaACT-16, blaNDM-1, blaTEM-1,***aph(6)-Id, aph(3′′)-Ib, tet(D), mph(A), rmtB, dfrA14, catA2 sul2*	*nlpI, terC, mrkA*
SAMN15904743	C	IncFIB(K),IncHI2,IncHI2A,IncR,IncX3,IncN3,Col(pHAD28,Col440I	**blaACT-16,blaNDM-1,blaCTX-M-13,blaOXA-1,blaTEM-1,blaSFO-1,blaSHV-12,***aph(6)-Id,aph(3′)-Ia,aac(3)-II,aadA2,armA,aac(6′)-Ib-cr,aph(3′′)-Ib,mcr-9,qacE,OqxA,OqxB, fosA,erm(B),msr(E),mph(A),mph(E),catB3,ARR-3,sul1,dfrA19,dfrA12*	*nlpI, terC, kpsM_K11*
SAMN21590478	C	IncFIB(pECLA),IncFII(pECLA),IncHI2,IncHI2A,IncN2,Col440I	**blaACT-16,blaNDM-1,blaOXA-1,blaTEM-1,blaCTX-M-15,** *aph(6)-Id,aadA1,aac(6′)-Ib-cr,aph(3′′)-Ib,aac(3)-IIa, OqxA,OqxB,fosA,catB3,floR,catA1,qnrB1,qnrS1,sul2,tet(A),tet(D),dfrA1,dfrA14*	*nlpI, terC*
SAMN33955250	C	IncFII(pECLA)/IncFIB(pECLA), IncFII(pKPX1), IncR,Col440I	**blaACT-16, blaNDM-1, blaTEM-1, blaOXA-1** *aph(3′)-Ia, aph(3′′)-Ibaph(6)-Id, aac(6′)-Ib3, aac(6′)-Ib-cr, ARR-3, fosA, mph(A), catB3, qacE, sul1, sul2, tet(D), OqxA, OqxB, qnrB19*	*nlpI, terC*
SAMN14669245	C	IncFIB(pECLA)/IncFII(pECLA),IncQ1,IncR,IncX3	**blaACT-16,blaDHA-1,blaOXA-1,blaTEM-1,blaNDM-5,***aph(6)-Id,aac(6′)-Ib-cr_2,aph(3′′)-Ib, qacE,OqxA,OqxB, fosA,mph(A),catB3,catA2,qnrB4,ARR-3,sul1,sul2,tet(D),dfrA14*	*nlpI, terC*

Supplementary Table S5. Distribution of the 55 ST182 *E. hormaechei* isolates into genomic clusters, continent and country of isolation, *bla*NDM variants and *bla*NDM-carrying plasmid types.

Characteristics	Cluster			Total
	A	B	C	
**Continent**				
Africa	4			4
Asia	11	2	4	17
Europe	12	4	1	17
North America	9	2	3	14
South America	1	2		3
**Total**	**37**	**10**	**8**	**55**
**Country**				
China	3			3
Colombia		2		2
Czech Republic	2			2
Germany	1			1
Greece			1	1
Guatemala	1			1
Hong Kong			1	1
India		1		1
Iran	1			1
Mexico	1			1
Myanmar	3			3
Netherlands	4	1		5
Pakistan	2			2
Philippines	1			1
Senegal	2			2
Singapore	1	1	1	3
Spain	3			3
Switzerland		1		1
Thailand			1	1
Togo	1			1
Tunisia	1			1
United Kingdom	2	2		4
USA	8	2	3	13
Viet Nam			1	1
**Total**	**37**	**10**	**8**	**55**
*bla*NDM	24	1	5	30
*bla*NDM-1	19	1	4	24
*bla*NDM-4	2			2
*bla*NDM-5	1		1	2
*bla*NDM-7	2			2
None	13	9	3	25
**Total**	**37**	**10**	**8**	**55**
*bla*NDM-carrying plasmid type	24	1	5	30
pGUE-NDM (IncFII)	2	1		3
pJN24NDM1 (IncN2)			1	1
pKOX_NDM-1 (IncFII)	8			8
pKPX-1 (Inc FII)			1	1
pM214_AC2 (IncA/C)	1			1
pNDM-HN380 (IncX3)	13		3	16
None	13	9	3	25
**Total**	**37**	**10**	**8**	**55**

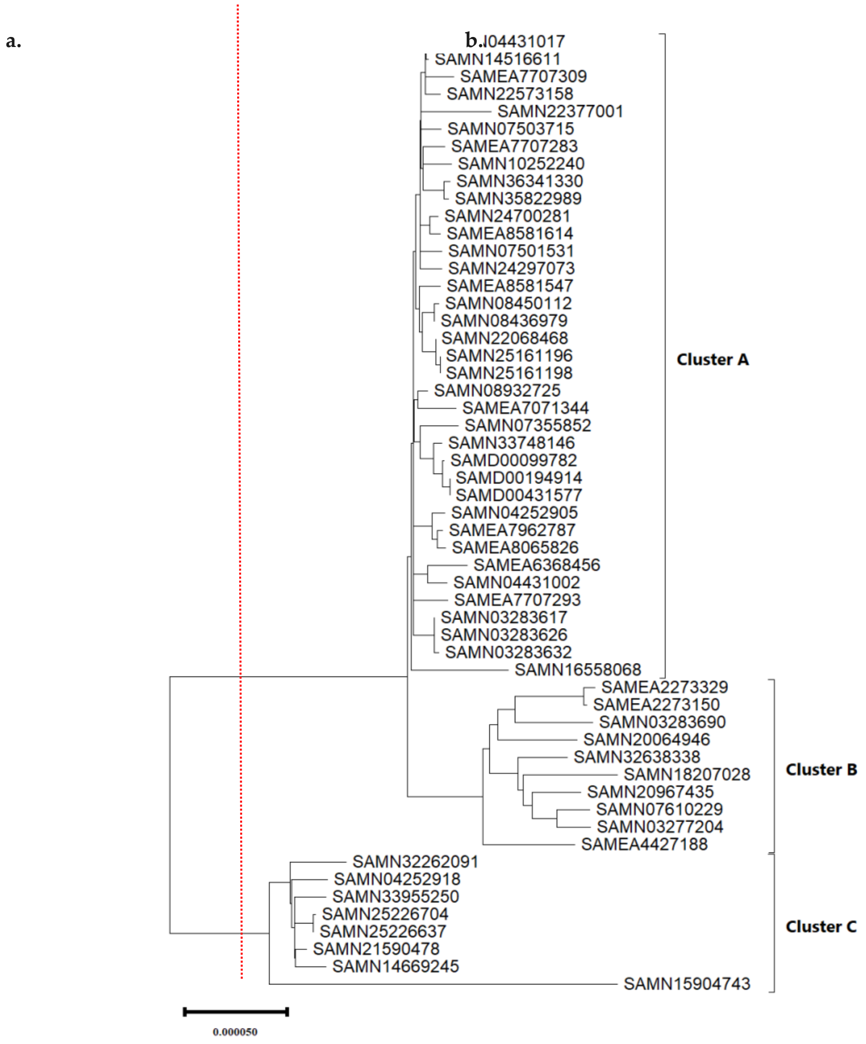

Supplementary Figure S1. Evolutionary relationships of ST182 *E.hormaechei* WGS assemblies. Singe-nucleotide polymorphisms (SNPs) were extracted using the Galaxy Server the aligned set of orthologous sites obtained by REALPHY. Evolutionary analyses were conducted by modules incorporated in MEGA11 [1-5]. The nucleotide sequences of the SNPs were aligned by MUSCLE [2]. The evolutionary distances were computed using the Kimura 2-parameter method [3] and are in the units of the number of base substitutions per site. This analysis involved 55 nucleotide sequences. Codon positions included were 1st+2nd+3rd+Noncoding. All ambiguous positions were removed for each sequence pair (pairwise deletion option). There were a total of 4554 positions in the final dataset. The evolutionary history was inferred using the Neighbor-Joining method [4,5]. The optimal tree is shown.

1. Tamura K., Stecher G., and Kumar S. (**2021**). MEGA 11: Molecular Evolutionary Genetics Analysis Version 11. *Molecular Biology and Evolution* https://doi.org/10.1093/molbev/msab120
2. Edgar RC. MUSCLE: multiple sequence alignment with high accuracy and high throughput. Nucleic Acids Res. 2004 Mar 19;32(5):1792-7. doi: 10.1093/nar/gkh340. PMID: 15034147; PMCID: PMC390337.
3. Kimura M. (**1980**). A simple method for estimating evolutionary rate of base substitutions through comparative stud.ies of nucleotide sequences. *Journal of Molecular Evolution* **16**:111-120.
4. Saitou N. and Nei M. (**1987**). The neighbor-joining method: A new method for reconstructing phylogenetic trees. *Molecular Biology and Evolution* **4**:406-425.
5. Felsenstein J. (**1985**). Confidence limits on phylogenies: An approach using the bootstrap. *Evolution* **39**:783-791.

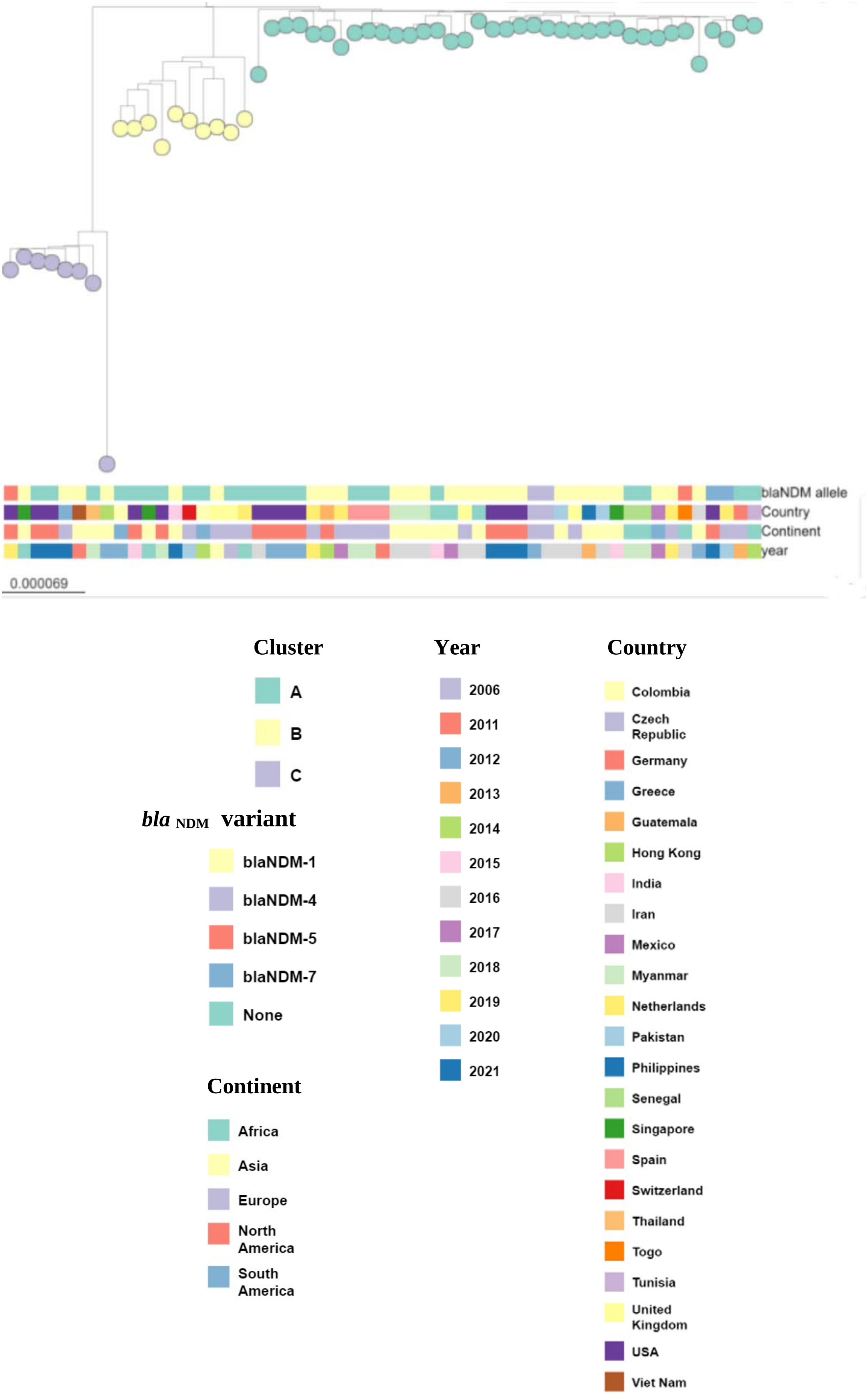

Supplementary Figure S2. Genomic clusters, *bla*NDM variants, country, continent and year of isolation of 55 ST182 *E. hormaechei* WGS assemblies.

a.

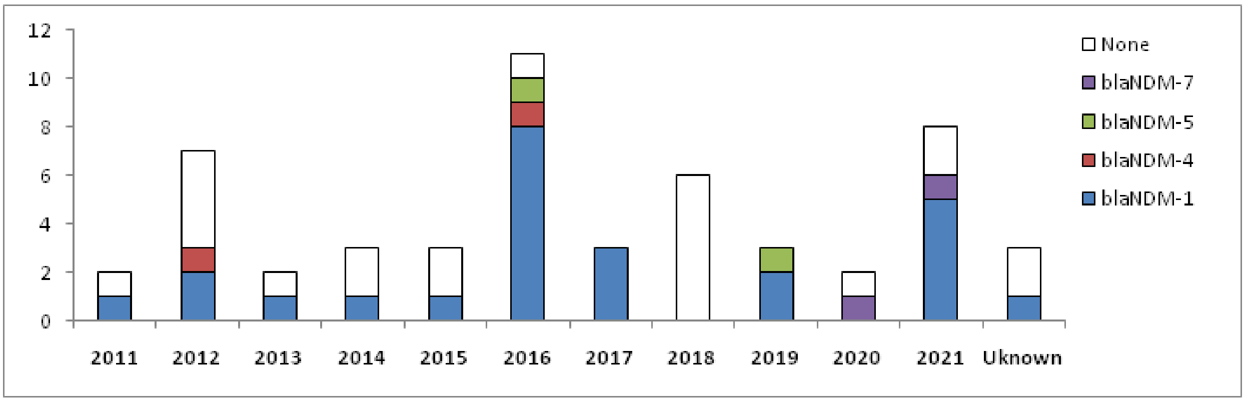

b.

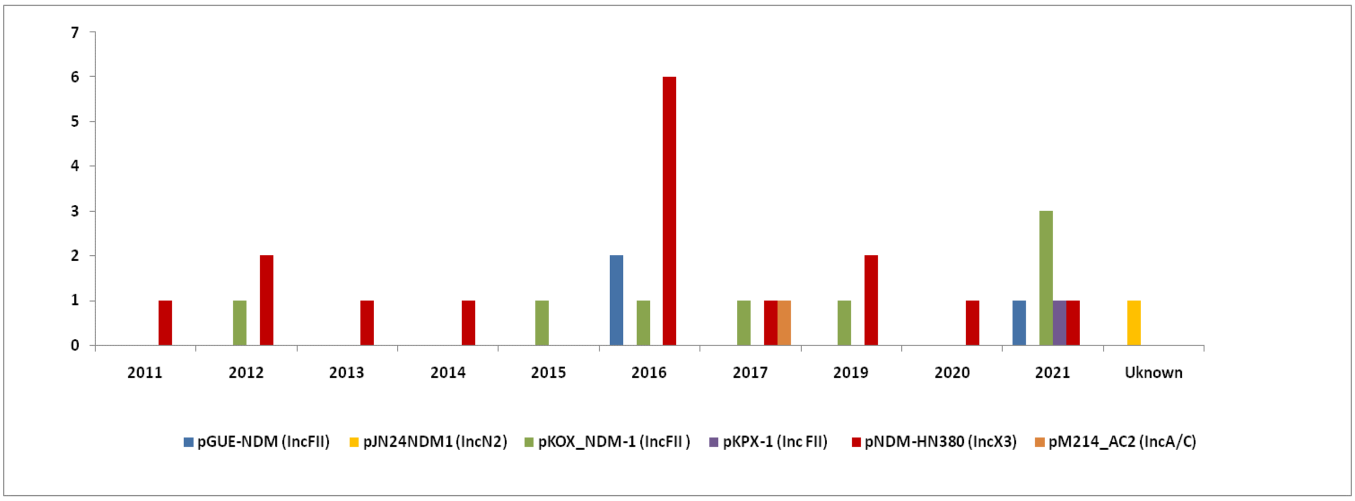

Supplementary Figure S3. Yearly distribution of ST182 *E. hormaechei*(a)*bla*NDM variants and (b) plasmid types of *bla*NDM-carrying WGS assemblies during 2011-2021.

4a. pNDM-HN380-like (IncX3) contigs

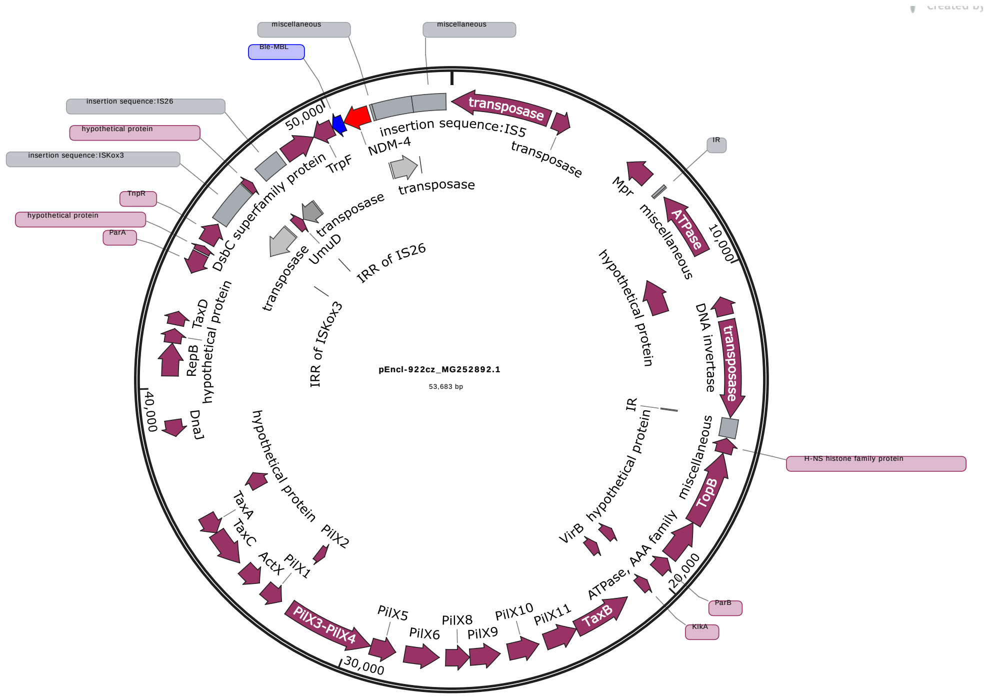

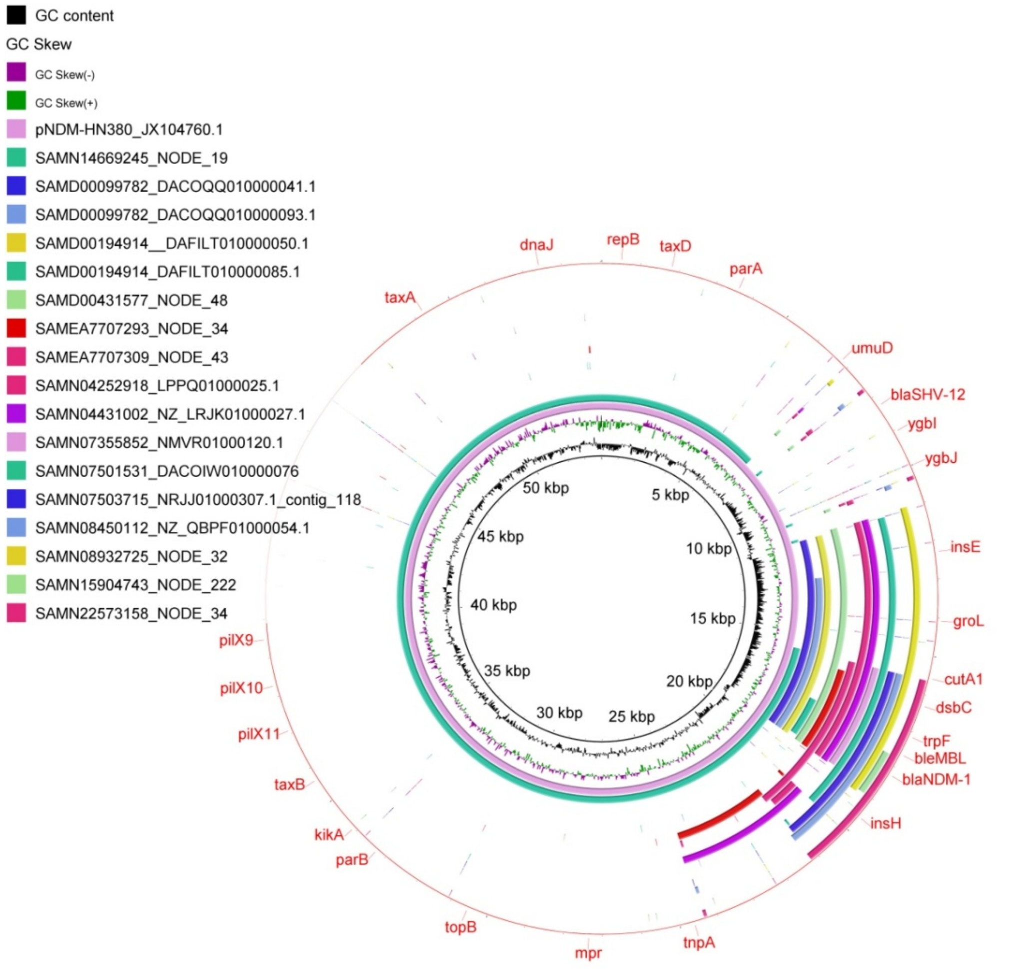

4b.pGUE-NDM-like (IncFII) contigs

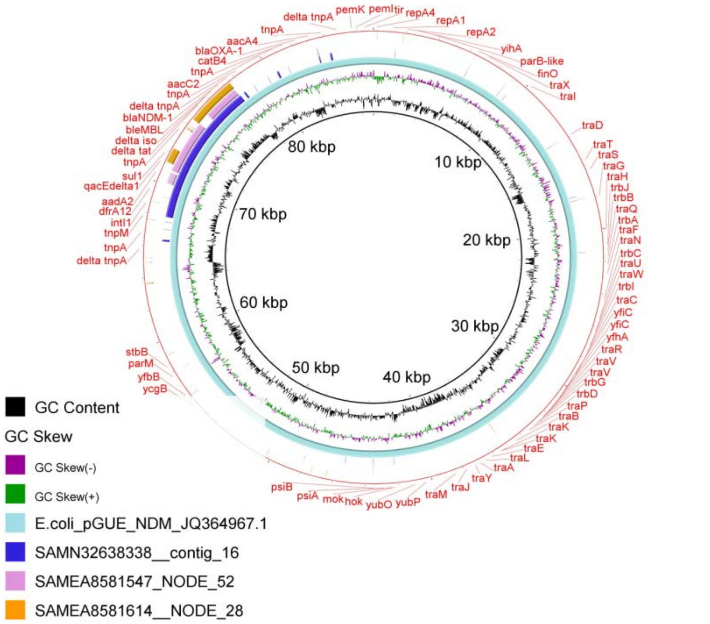

4c. pKOX_NDM-1-like (IncFII) contigs

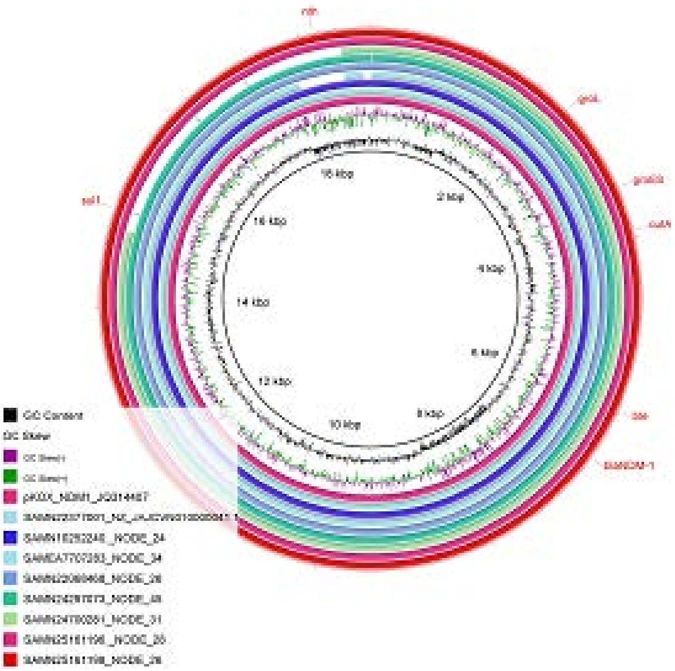

4d. pKPX-1-like (IncFII) contigs

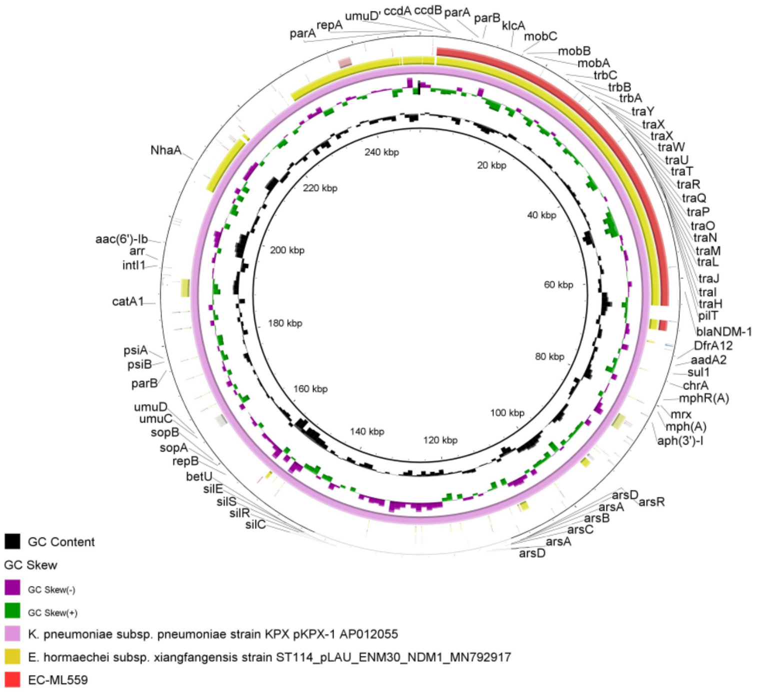

4e. pJN24NDM1-like (IncN2) contigs

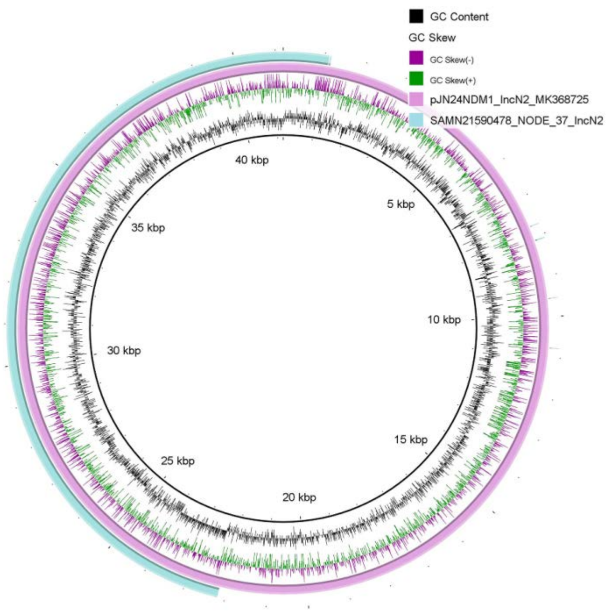

4f. pM214_AC2-like (IncA/C2) contigs

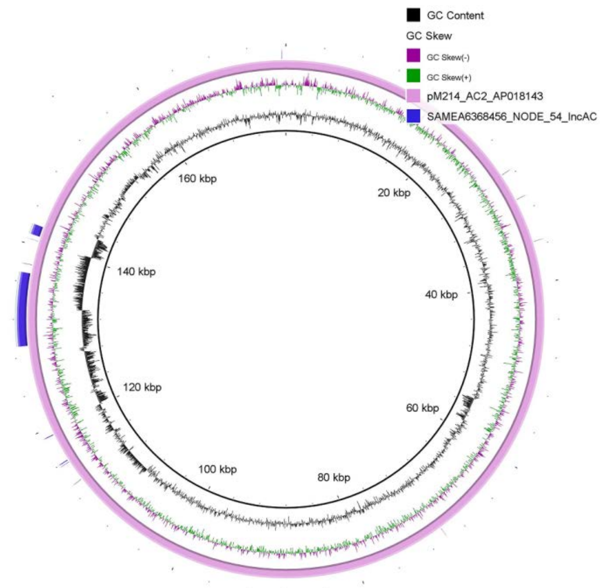

Supplementary Figure S4. BlastN comparisons, of the nucleotide sequences of *bla*$_{NDM}$-harbouring contigs: (4a)pNDM-HN380-like (IncX3) contigs, (4b)pGUE-NDM-like (IncFII) contigs, (4c) pKOX_NDM-1-like (IncFII) contigs,(4d) pKPX-1-like (IncFII) contigs, 4E) pJN24NDM1-like (IncN2) contigs, 4F) pM214_AC2-like (IncA/C2) contigs.
